# Prevalence of urinary incontinence and pelvic floor muscle dysfunction in primiparae two years after cesarean section: cross-sectional study

**DOI:** 10.1590/S1516-31802013000100019

**Published:** 2013-04-01

**Authors:** Angélica Mércia Pascon Barbosa, Gabriela Marini, Fernanda Piculo, Cibele Vieira Cunha Rudge, Iracema Mattos Paranhos Calderon, Marilza Vieira Cunha Rudge

**Affiliations:** I PhD. Professor, Department of Gynecology and Obstetrics, Faculdade de Medicina de Botucatu, Universidade Estadual Paulista (FMB-Unesp), Botucatu, São Paulo, Brazil.; II Doctoral Student. Department of Gynecology and Obstetrics, Faculdade de Medicina de Botucatu, Universidade Estadual Paulista (FMB-Unesp), Botucatu, São Paulo, Brazil.; III Master Degree Student. Department of Gynecology and Obstetrics, Faculdade de Medicina de Botucatu, Universidade Estadual Paulista (FMB-Unesp), Botucatu, São Paulo, Brazil.; IV MD, PhD. Professor, Department of Gynecology and Obstetrics, Faculdade de Medicina de Botucatu, Universidade Estadual Paulista (FMB-Unesp), Botucatu, São Paulo, Brazil.

**Keywords:** Cesarean section, Pelvic floor, Postpartum period, Pregnancy, Urinary incontinence, Cesárea, Diafragma da pelve, Período pós-parto, Gravidez, Incontinência urinária

## Abstract

**CONTEXT AND OBJECTIVE:**

There is uncertainty in the literature regarding the theory that obstetric events and pelvic floor injuries give rise to lower risk of subsequent urinary incontinence among women delivering via cesarean section than among women delivering vaginally. The objective of this study was to assess the two-year postpartum prevalence of urinary incontinence and pelvic floor muscle dysfunction and the factors responsible for them.

**DESIGN AND SETTING:**

Cross-sectional study, conducted in a public university.

**METHODS:**

220 women who had undergone elective cesarean section or vaginal childbirth two years earlier were selected. Their urinary incontinence symptoms were investigated, and their pelvic floor muscle dysfunction was assessed using digital palpation and a perineometer.

**RESULTS:**

The two-year urinary incontinence prevalences following vaginal childbirth and cesarean section were 17% and 18.9%, respectively. The only risk factor for pelvic floor muscle dysfunction was weight gain during pregnancy. Body mass index less than 25 kg/m^2^ and normal pelvic floor muscle function protected against urinary incontinence. Gestational urinary incontinence increased the risk of two-year postpartum urinary incontinence.

**CONCLUSION:**

Gestational urinary incontinence was a crucial precursor of postpartum urinary incontinence. Weight gain during pregnancy increased the subsequent risk of pelvic floor muscle dysfunction, and elective cesarean section did not prevent urinary incontinence.

## RESUMO

**CONTEXTO E OBJETIVO:**

É ainda controversa na literatura a teoria de que eventos obstétricos e traumas no assoalho pélvico representariam menor risco para mulheres submetidas ao parto cesárea do que para aquelas submetidas a parto vaginal, no tocante a subsequente incontinência urinária. O objetivo do estudo foi avaliar a prevalência de incontinência urinária e disfunção muscular do assoalho pélvico dois anos após o parto e os fatores responsáveis por elas.

**TIPO DE ESTUDO E LOCAL:**

Estudo transversal conduzido em universidade pública.

**MÉTODOS:**

Foram selecionadas 220 mulheres dois anos após parto cesáreo eletivo ou parto vaginal. Foram avaliados sintomas de incontinência urinária e disfunção muscular do assoalho pélvico por palpação digital e perineômetro.

**RESULTADOS:**

A prevalência de incontinência urinária dois anos após parto vaginal e cesárea foi de 17% e 18,9% respectivamente. O único fator de risco para disfunção muscular do assoalho pélvico foi o ganho de peso durante a gestação. Índice de massa corporal inferior a 25 kg/m^2^ e disfunção muscular do assoalho pélvico normal foram fatores de proteção contra incontinência urinária. Incontinência urinária na gestação aumentou o risco de incontinência urinária dois anos pós-parto.

**CONCLUSÃO:**

Incontinência urinária gestacional foi um precursor crucial de incontinência urinária pós-parto. O ganho de peso durante a gestação aumentou o risco posterior de disfunção muscular do assoalho pélvico e o parto cesárea eletivo não foi uma ação de prevenção para a incontinência urinária.

## INTRODUCTION

Elective cesarean section without labor is a common procedure in Brazil, accounting for more than 40% of births. In the past, this procedure was thought to protect against urinary incontinence.^1^ However, there are doubts as to whether an elective cesarean section confers an independent protective effect against urinary incontinence. Incontinence rates have not been correlated with delivery method, but elective cesarean sections without attempting labor have been associated with a significantly lower prevalence of postpartum urinary incontinence.^2^

It is questionable whether cesarean section delivery can prevent pelvic floor injury,^3^^,^^4^^,^^5^ but recent data have suggested that this protective effect is less pronounced and that gestational urinary incontinence appears to be the most important predictive factor for developing postnatal urinary incontinence.^6^^,^^7^^,^^8^ The risk of urinary incontinence has been found to be higher among women who had only experienced cesarean delivery than among nulliparous women, and even higher among those who had only delivered vaginally.^9^

In women without urinary incontinence, the pelvic floor muscles contract simultaneously with or immediately before increases in abdominal pressure as an unconscious involuntary automatic co-contraction.^10^ A variety of methods have been used in clinical practice and research to evaluate pelvic floor muscle function and strength, but no single measurement tool provides a full picture of pelvic floor muscle strength or function with sufficiently demonstrated responsiveness, reliability and validity to allow it to measure the automatic actions of the pelvic floor muscles in real-life situations.^11^

There is uncertainty about the relationship between obstetric events and pelvic floor injury and about whether women delivering by cesarean section are at lower risk of subsequent urinary incontinence than are women who deliver vaginally.^12^^,^^13^^,^^14^

## OBJECTIVE

The aim of this cross-sectional study was to assess the two-year postpartum prevalence of urinary incontinence and pelvic floor muscle dysfunction among primiparae following cesarean section, and to determine the factors responsible for two-year postpartum urinary incontinence and pelvic floor muscle dysfunction.

## METHODS

The Institutional Review Board of the Faculdade de Medicina de Botucatu, Universidade Estadual Paulista (FMB-Unesp), São Paulo, Brazil, granted approval for this study. This was a cross-sectional study including all of the women who delivered between June 1, 2008, and February 28, 2009, and who were eligible for a postpartum interview on urinary incontinence and persistent pelvic floor injury. A total of 832 women were identified using data from the Health Department Registration System.

A simple telephone questionnaire was used to recruit primiparous women who were between 20 and 30 years old when they delivered; delivered at term (by means of either elective cesarean section or spontaneous vaginal childbirth); and delivered an infant with a birth weight lower than 4 kg. The exclusion criteria were previous miscarriage, pelvic or abdominal surgery, previous urogynecological surgery and chronic illnesses such as diabetes mellitus, hypertension, chronic rheumatoid disease or neurological disorders. From these inclusion and exclusion criteria, 355 women were recruited (40.2%).

Using pelvic floor strength, an a of 0.05 and a b of 0.2, the required sample size for this study was 106 women with cesarean section and 106 with vaginal childbirth.^15^

Two years postpartum, all of the women were interviewed in person regarding their urinary incontinence symptoms and the prevalence of urinary incontinence before and during the pregnancy and in the two years after the “index birth”. All of the women were questioned by the same research. They were also asked whether they had consulted a physician regarding their urinary incontinence and whether they desired further evaluation and treatment.

The obstetric and maternal data relevant to the “index birth” were retrieved from the hospital records. The following maternal, fetal and obstetric parameters were assessed: maternal age, weight and height; weight gain during pregnancy; gestational age at delivery; delivery method (cesarean section or vaginal childbirth); and birth weight. The body mass index, i.e. the ratio of present weight in kilograms divided by height in meters squared (kg/m^2^), was also calculated.

A conventional clinical evaluation was then performed, and pelvic floor contraction strength was assessed by means of digital examination, which was performed with the knees semi-flexed. A score of between 0 and 3 was given, as described by Amaro et al.^16^ We considered digital examination scores of less than 3 to be abnormal.

Vaginal manometry (perineometry) was performed using a vaginal latex-sensor perineometer, with the woman in the same position used for the pelvic floor contraction assessment. Manometric values higher than 33.6 mmHg were considered normal.^15^ The women answered the Questionnaire for Urinary Incontinence Diagnosis^17^ and the International Consultation on Incontinence Questionnaire - Short Form (ICIQ-SF), which had been translated into Portuguese and validated among female Brazilian patients complaining of urinary incontinence.^18^ The questionnaire investigated the following aspects of pregnancy and childbirth: maternal weight gain, newborn weight, whether the mother had entered into partum labor, postpartum complications, involuntary urine loss (under circumstances such as coughing, sneezing, laughing, lifting weights, squatting or walking, and/or when feeling a strong urge to urinate, being in contact with water, hearing running water and being exposed to low temperatures), during pregnancy and the postpartum period.

The data were analyzed using two different methods. Student’s t test was used for comparing the means, and the Z test was used for investigating the differences between proportions. The adjusted odds ratios (ORs) and their 95% confidence intervals (95% CIs) were calculated using the Mantel-Haenszel c^2^ test. For the logistic regression analysis, urinary incontinence and pelvic floor muscle dysfunction were used as the reference variables. The statistical calculations were performed using the SPSS 12.0 (Statistical Package for Social Sciences) and SAS 8.02 software programs. The statistical significance level was set at P < 0.05.

## RESULTS

The population characteristics showed no significant differences in maternal age, gestational age at delivery, weight gain during pregnancy or birth weight. Two years after delivery, the prevalence of urinary incontinence was 17% following vaginal childbirth and 18.9% following cesarean section. No significant differences were found between the cesarean section and vaginal childbirth groups regarding the incidence of either urinary incontinence or pelvic floor muscle dysfunction (Table 1).

**Table 1.** Characteristics of the study population and prevalence of urinary incontinence and pelvic floor strength dysfunction

Variables	Vaginal delivery n = 106		Cesarean section n = 106		P value
	n (%)	? **SD**	n (%)	? **SD**	
Age (y)		25.04 ? 3.18		24.57 ? 3.17	0.291
Gestational age at delivery (w)		39.86 ? 1.31		39.68 ? 1.32	0.323
Weight gain during pregnancy (kg)		14.67 ? 3.94		14.59 ? 4.10	0.892
Birth weight (g)		3119.05 ? 421.17		3141.41 ? 464.64	0.714
Pelvic floor muscle digital palpation - abnormal	75 (70.8)		74 (69.8)		0.88
Pelvic floor muscle perineometer - abnormal	61 (57.5)		60 (56.6)		0.89
Gestational urinary incontinence	37 (34.9)		34 (32.1)		0.66
Two-year postpartum urinary incontinence	18 (17.0 )		20 (18.9)		0.72

SD = standard deviation.

There was an increased risk of subsequent pelvic floor muscle dysfunction with increasing weight gain during pregnancy (OR = 1.3 and 95% CI = 1.1-1.4 for digital palpation; OR = 1.2 and 95% CI = 1.0-1.3 for perineometry) (Table 2).

**Table 2.** Estimates obtained from the multivariate logistic regression model for the risk of pelvic floor muscle dysfunction two years after vaginal delivery or cesarean section

Variables	Digital palpation n = 212			Perineometer n = 212		P value
	OR	95% CI	P value	OR	95% CI	
Age (y)	0.98	0.889-1.081	0.6933	1.065	0.969-1.170	0.1923
Gestational age at delivery (w)	1.093	0.903-1.323	0.3591	1.133	0.946-1.358	0.0743
Weight gain during pregnancy (kg)	1.301	1.153-1.468	< 0.0001^*^	1.210	1.089-1.344	0.0004^*^
Birth weight (g)	1.000	0.999-1.001	0.8018	1.001	1.000-1.001	0.1300
Body mass index £ 25	0.941	0.859-1.031	0.1904	0.999	0.918-1.087	0.9800
Delivery method - vaginal or cesarean	1.025	0.541-1.945	0.1589	1.018	0.560-1.850	0.1524

*P < 0.05; OR = odds ratio; CI = confidence interval.

The patients with body mass indices £ 25 kg/m^2^ and normal pelvic floor muscle strength two years after delivery were less likely to complain of urinary incontinence (OR = 0.8, 95% CI = 0.7-0.9 and OR = 0.1, 95% CI = 0.03-0.8, respectively). The women with gestational urinary incontinence were more likely to describe urinary incontinence symptoms two years after delivery (OR = 8.6, 95% CI = 3.0-24.3). The delivery method was not a risk factor for urinary incontinence two years after the “index birth” (Table 3).

**Table 3.** Estimates obtained from the multivariate logistic regression model for the risk of urinary incontinence two years after vaginal delivery or cesarean section

Variables	Urinary incontinence		
	OR	95% CI	P value
Age (y)	0.885	0.771-1.017	0.0851
Gestational age at delivery (w)	1.198	0.879-1.632	0.2519
Birth weight (g)	1.000	0.999-1.001	0.4034
Body mass index two years after delivery £ 25	0.874	0.775-0.985	0.0269^*^
Gestational urinary incontinence	8.675	3.027-24.324	< 0.0001^*^
Delivery method - vaginal or cesarean	0.675	0.282-1.617	0.3782
Pelvic floor muscle - normal	0.184	0.038-0.895	0.0360^*^

^*^P < 0.05; OR = odds ratio; CI = confidence interval.

## DISCUSSION

The present study found that cesarean sections did not protect against urinary incontinence two years after childbirth in Brazil, a country in which 40% of deliveries are performed by means of elective cesarean section.^19^

Our results are consistent with those of Viktrup,^20^ who followed 278 primiparae for five years postpartum and found no statistically significant associations between first birth via cesarean section and urinary incontinence at follow-up. Zhu et al.^21^ evaluated the prevalence and risk factors for urge urinary incontinence among adult Chinese women and found that parity and the mode of delivery were not risk factors.

Elective cesarean section seems to have a limited protective effect that appears to weaken with time. Vaginal delivery in itself is neither a sufficient nor a necessary condition for urinary incontinence in most women. By inference, cesarean section is not sufficient to prevent all cases of urinary incontinence.^22^^,^^23^ It has been assumed that cesarean section delivery protects against urinary incontinence, and several studies on postpartum and general populations have confirmed this association. However, Herrmann et al.^24^ found no significant correlation between the incidence of stress urinary incontinence and the mode of delivery.

In a longitudinal cohort investigation of urinary incontinence in a postpartum population large enough to examine the effect of delivery methods over time, McArthur et al.^25^ found that delivery exclusively by means of cesarean section reduced the odds of developing persistent urinary incontinence by half. Even among this group, however, the prevalence of persistent urinary incontinence was still relatively high (14%). This value was quite similar to our results.

Our analysis on first childbirths among 212 women showed that the risk of urinary incontinence two years postpartum was raised considerably by urinary incontinence during pregnancy (OR = 8.6, 95% CI = 3.02-24.32) and that childbirth-induced urinary incontinence was not preventable by cesarean section. In Brazil, cesarean section is far more common than recommended by the World Health Organization and may seem to offer a potential means to prevent urinary incontinence. Our results confirmed the data of Foldspang et al.^26^ and Eason et al., ^7^ who found that urinary incontinence before delivery roughly doubles the likelihood of urinary incontinence postpartum, regardless of whether the delivery is vaginal or via cesarean section. Occurrences before delivery are an important risk factor for urinary incontinence afterwards and later in life. These protective data confirm that urinary incontinence beginning during pregnancy is neither trivial nor transient. They indicate that there is a significant risk of persistent urinary incontinence, even among women who deliver by cesarean section. Our results also confirm some studies that have found pregnancy-induced incontinence to be one of the strongest predictors of postpartum incontinence, regardless of the delivery route.^27^^,^^28^

The present data, which demonstrate similar pelvic floor muscle dysfunction two years after cesarean section and after vaginal childbirth, suggest that childbirth-induced urinary incontinence is not preventable by elective cesarean section. Identifying women at high risk of delivery-related pelvic floor trauma should be a priority for future research in this field.^22^ The current evidence does not support routine use of elective cesarean section to prevent urinary incontinence, and the delivery mode should continue to be dictated by obstetric considerations. This interpretation of our results is in agreement with Casey et al.,^29^ who reported: “childbirth-induced pelvic floor injury does not appear to us to be easily preventable by modifying obstetric practice.” Even the women with elective cesarean sections (in our population) showed levels of urinary incontinence and pelvic floor muscle dysfunction similar to those of the women who delivered vaginally.

Greater weight gain during pregnancy was associated with an increased risk of postpartum pelvic floor muscle dysfunction (OR = 1.3, 95% CI = 1.1-1.4 and OR = 1.2, 95% CI = 1.08-1.34). In addition, a body mass index £ 25 kg/m^2^ two years after delivery was a protective factor against urinary incontinence (OR = 0.8, 95% CI = 0.77-0.98), as has also been reported by other recent studies.^30^^,^^31^

Eftekhar et al.^2^ found that the prevalence of urinary incontinence was associated with a high birth weight (P = 0.00, c^2^ = 25.5). The effect of high birth weight is seen in the extra weight borne by the lower abdominal organs during pregnancy, in addition to the size of the infant that has to pass through the delivery canal. Our results do not confirm that birth weight has an influence on urinary incontinence two years after delivery, and this was probably because only deliveries with birth weights lower than 4000 g were included in this study.

## CONCLUSION

The prevalence of urinary incontinence and pelvic floor muscle dysfunction was unrelated to delivery method, and cesarean section did not protect against urinary incontinence two years after delivery. Urinary incontinence during pregnancy is a crucial precursor of urinary incontinence two years postpartum. Weight gain during pregnancy increased the risk of subsequent pelvic floor muscle dysfunction, while a body mass index £ 25 kg/m^2^ two years after delivery prevented urinary incontinence.

Further research on a larger group is needed in order to make definitive statements regarding the effects of delivery method on urinary incontinence and pelvic floor dysfunction two years postpartum.
